# The Benefits of Anthocyanins against Obesity-Induced Inflammation

**DOI:** 10.3390/biom12060852

**Published:** 2022-06-20

**Authors:** Chanya Ngamsamer, Jintana Sirivarasai, Nareerat Sutjarit

**Affiliations:** 1 Doctor of Philosophy Program in Nutrition, Faculty of Medicine, Ramathibodi Hospital and Institute of Nutrition, Mahidol University, Bangkok, 10400, Thailand; chanya.ngm@student.mahidol.edu; 2 Graduate Program in Nutrition, Faculty of Medicine, Ramathibodi Hospital, Mahidol University, Bangkok, 10400, Thailand; jintana.sir@mahidol.ac.th

**Keywords:** obesity, inflammation, meta-inflammation, anthocyanin, antioxidant, anti-inflammation

## Abstract

Obesity has become a serious public health epidemic because of its associations with chronic conditions such as type 2 diabetes mellitus, hypertension, cardiovascular disease, and cancer. Obesity triggers inflammation marked by the secretion of low-grade inflammatory cytokines including interleukin-6, C-reactive protein, and tumor necrosis factor-α, leading to a condition known as “meta-inflammation”. Currently, there is great interest in studying the treatment of obesity with food-derived bioactive compounds, which have low toxicity and no severe adverse events compared with pharmacotherapeutic agents. Here, we reviewed the beneficial effects of the bioactive compounds known as anthocyanins on obesity-induced inflammation. Foods rich in anthocyanins include tart cherries, red raspberries, black soybeans, blueberries, sweet cherries, strawberries and Queen Garnet plums. These anthocyanin-rich foods have been evaluated in cell culture, animal, and clinical studies, and found to be beneficial for health, reportedly reducing inflammatory markers. One factor in the development of obesity-related inflammation may be dysbiosis of the gut microbiome. Therefore, we focused this review on the in vitro and in vivo effects of anthocyanins on inflammation and the gut microbiota in obesity.

## 1. Introduction

Obesity, which has been rising in prevalence worldwide over the past several decades, is now considered a public health epidemic. Obesity for adults is defined as a body mass index of ≥30 kg/m^2^ in Western populations, while the current Asia-Pacific guidelines recommend using a lower body mass index of ≥25 kg/m^2^ [1]. Obesity is linked to metabolic conditions such as hyperglycemia and dyslipidemia, which are well-known risk factors for developing chronic noncommunicable diseases, including hypertension, type II diabetes mellitus (T2DM), cancer, and cardiovascular disease (CVD) [2]. Individuals with obesity exhibit chronic low-grade systemic inflammation, which is characterized by increased secretion of pro-inflammatory cytokines from adipose tissue, made dysfunctional by the excessive accumulation of fat into the circulation; these cytokines include interleukin-6 (IL-6), C-reactive protein (CRP) and tumor necrosis factor-α (TNF-α) [3].

Generally, treatments for obesity include lifestyle modification and pharmacotherapy aimed at reducing body weight and alleviating inflammation [4]. Clinical guidelines suggest a combination of lifestyle modification and pharmacotherapy [5]. However, such medicines are associated with severe adverse events such as mental disorders and stroke, and they are quite expensive [6,7]. Currently, there are many studies aimed at treating obesity through dietary lifestyle modifications. Bioactive chemicals, particularly those that are plant or animal-based compounds, have the potential to diminish the prevalence of chronic disorders [8]. Some bioactive compounds are also classified as phytochemicals. Phytochemicals such as polyphenols have antioxidant, anti-inflammatory, anti-obesity, and anti-aging properties [9,10].

## 2. Human Obesity: Its Causes and Consequences

The World Health Organization reports that “obesity has almost tripled globally since 1975”, highlighting that obesity has become a global epidemic. In 2016, more than 1.9 billion of the world’s population were overweight; of these, over 650 million individuals were classified as obese [11]. More importantly, the obesity epidemic is increasingly affecting children, with an estimated 38.2 million children under the age of 5 years suffering from obesity in 2019 [12]. The cornerstone of the disease of obesity is an energy imbalance that occurs when energy consumption, particularly excessive fat and sugar intake, exceeds energy expenditure. Currently, many individuals with extreme obesity lead a sedentary lifestyle and eventually become bedridden. In fact, the continuous increase in noncommunicable diseases such as T2DM, CVD, hepatic steatosis, neurodegenerative diseases, biliary diseases, and certain cancers are directly linked to the increased prevalence of human obesity [13]. Importantly, these obesity-related diseases also result in a shorter life expectancy and premature death.

## 3. Obesity-Induced Inflammation

**Figure 1:**
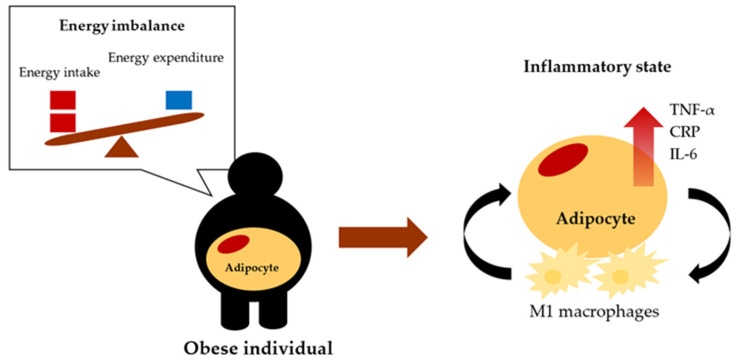
Hallmark of meta-inflammation. The major cause of obesity is a positive energy balance between energy intake and energy expenditure leading to meta-inflammation, a chronic low-grade inflammatory condition.

Obesity is associated with inflammation through increased pro-inflammatory cytokine secretion resulting from inflammatory responses and altered metabolic homeostasis. “Meta-inflammation” is a term used to describe chronic low-grade inflammation as a response to obesity [13]. In obesity, excessive accumulation of fat in adipocytes causes adipose tissue dysfunction [14]. Compared with non-obese individuals, the dysfunctional adipose tissues in rodent models of obesity and humans with obesity secrete lower amounts of adiponectin, which exerts an anti-inflammatory effect on the liver, skeletal muscle, and adipose tissue. These dysfunctional tissues simultaneously secrete increased levels of pro-inflammatory cytokines such as CRP, IL-6 and TNF-α [15], which disrupt the metabolic state required to maintain immune homeostasis. Innate immune cells secrete several cytokines and acute-phase proteins to the sites of inflammation [3,16]. In people with obesity, there is a phenotypic switching from M2 macrophages to M1 macrophages in the adipose tissues. M2 macrophages, which are anti-inflammatory, play a role in the maintenance of tissue and are typical in the adipose tissues of lean individuals. By contrast, M1 macrophages produce pro-inflammatory cytokines and thus contribute to the development of insulin resistance [17]. An increase in M1 macrophages, which form a structure around adipocytes and release pro-inflammatory mediators such as IL-1, IL-6, IL-12, TNF-α, and chemokines, is part of the alteration in immune cell profile that is characteristic of obesity (Figure 1). As a result, tissue damage to the liver, colon, and arterial walls may occur. One possible underlying mechanism of obesity-induced inflammation is the activation of toll-like receptors (TLRs) of the innate immune system in obese patients, particularly TLR4, which involves the activation of transcription factors such as nuclear factor kappa B (NF-κB) and the production of pro-inflammatory markers [18,19].

In patients with prolonged obesity, there may be a switch from the innate immune response to the adaptive immune response, leading to the onset of obesity-associated chronic diseases. Thus, obesity-related chronic diseases can be prevented or delayed by preventing long-term obesity [16]. Below we review the nutraceutical and pharmaceutical effects of anthocyanins that might be beneficial for treating obesity-related inflammation.

## 4. Anthocyanins

Anthocyanins are water-soluble pigments within the phenol class of compounds. They are abundant in nature, and can be found in vegetables, fruits, and flowers. Anthocyanin pigments in plants occur in the form of glycosides. All anthocyanins play a significant role in preventing chronic noncommunicable diseases.

### 4.1. Chemistry of Anthocyanins

**Figure 2:**
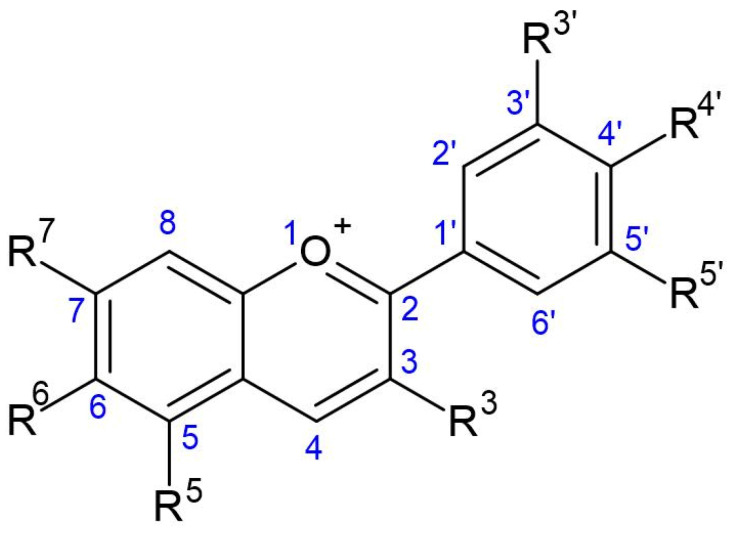
Fundamental structure of anthocyanin. (Drawn using the tool ACD/ChemSketch, version 2021.2.1, Advanced Chemistry Development, Inc., Toronto, ON, Canada).

**Figure 3:**
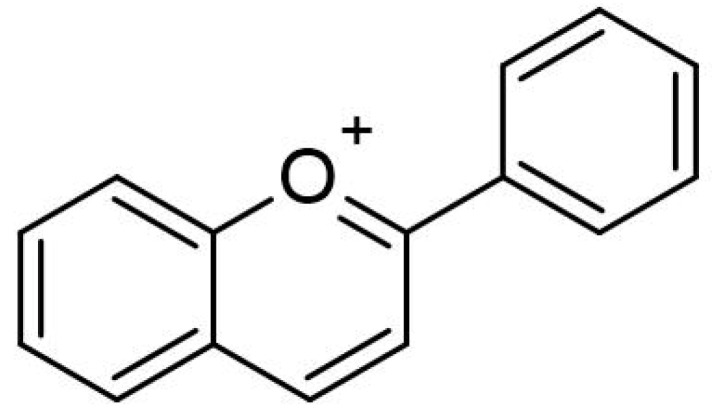
Two-dimensional structure of a flavylium ion. (Drawn using the tool ACD/ChemSketch, version 2021.2.1, Advanced Chemistry Development, Inc., Toronto, ON, Canada).

Anthocyanins are considered to be flavonoids, although they have a positive charge at the oxygen atom of the C-ring of the fundamental flavonoid structure. Such a compound is also called the flavylium (2-phenylchromenylium) ion. The general molecular structure of anthocyanins is shown in Figure 2. Anthocyanins are derived from flavanols, but with a flavylium ion lacking a ketone oxygen at the 4-position (Figure 3) [20]. The empirical formula for the flavylium ion of anthocyanin is C_15_H_11_O^+^ with a molecular weight of 207.24724 g/mol [21].

### 4.2. Bioavailability of Anthocyanins

The majority of bioactive compounds in functional foods have been linked to lower risks of chronic conditions including CVD and T2DM [8]. Some bioactive compounds, such as polyphenols, are also phytochemicals. Polyphenolic compounds have antioxidant, anti-inflammatory, anti-obesity, and anti-aging properties [9,10]. Apart from the overall content and biological functions of bioactive components, bioavailability is also an important factor. The term “bioavailability” is defined by the US Food and Drug Administration as the rate and extent to which an active ingredient is absorbed from a drug product and becomes available at the site of action, and includes metabolism and excretion [22]. The anthocyanins have exhibited low bioavailability in animal studies, as indicated by low systemic concentrations [23,24,25]. Examinations of plasma after intake of anthocyanin-rich foods such as blackcurrant juice, red wine, and strawberries, have revealed that anthocyanins have limited bioavailability in humans as well [26,27,28]. Previous research in both humans and animals has established that the low bioavailability of anthocyanins is attributable to their limited absorption into the circulation and significant elimination in urine and feces.

In contrast, a study on the phytochemical uptake following human consumption of the Montmorency tart cherry, which is rich in anthocyanins, has shown that anthocyanin metabolites are most bioavailable in the plasma at 1–2 hours post consumption, indicating that anthocyanins might be rapidly absorbed [29]. Moreover, a literature survey on the bioavailability of anthocyanins and anthocyanin-containing foods among humans by Manach et al. (2005), found that the bioavailability of anthocyanins is generally poor, although they may be rapidly absorbed at first. Multiple authors, as cited by Manach, et al. (2005), note that anthocyanins are absorbed rapidly in a manner consistent with stomach absorption, but the absorption efficiencies were very poor with plasma concentrations approximately six orders of magnitude lower than the ingested dose. Furthermore this small absorbed fraction was rapidly eliminated via the urine. [30]. However, there are novel technologies that can be utilized to increase anthocyanin bioavailability, as discussed below.

### 4.3. Stability of Anthocyanins

The stability of anthocyanin pigments are affected by pH, light, temperature, and structure [20]. Anthocyanins change color in response to different pH levels. Under acidic conditions, anthocyanins may appear as the red-colored flavylium ion, while under alkaline conditions, they change to the blueish color of quinoidal bases [31]. Temperature also affects anthocyanin color. In higher solution temperatures, anthocyanins can become less stable due to coupled oxidation reactions of peroxidase and hydrogen peroxide (H_2_O_2_) [32]. The food industry offers many different fruit and vegetable products that are rich in anthocyanins, such as juices and supplements. However, industrial anthocyanin-containing products are often subjected to thermal processing and other common food processing methods that can decrease stability and total anthocyanin content [33]. In the last decade, novel food-processing technologies such as high pressure processing (HPP), high hydrostatic pressure (HHP), and pulsed electric fields (PEF) have been developed that may help prevent the loss of color, total anthocyanin content, and potential health benefits of anthocyanins [34]. To prevent the loss of potential health benefits specifically, we suggest the application of the following novel technologies in food processing: A previous study reported that HPP treatment of blueberry juice led to minor changes in ascorbic acid, total phenolics, anthocyanin stability, and total antioxidant capacity compared with fresh blueberry juice because the pressure–time conditions were maximized at a maximum temperature of 42 °C [35]. Encapsulation technology can also protect a bioactive molecule such as anthocyanin from the effects of oxygen, light, or other factors. Spray-drying is a common technique used for the encapsulation of compounds [36]. An extract of cultivated purple flesh potato (PFPE) was encapsulated by spray-drying with maltodextrin (MD) as the encapsulating agent. The constant degradation rate and bioavailability of anthocyanins within an in vitro gastrointestinal digestion model are significantly higher in PFPE-MD than in non-encapsulated PFPE because powder encapsulation protects the stable colorant and health benefits of anthocyanins [37]. Kanokpanont et al. (2018) demonstrated that anthocyanin-encapsulated alginate/chitosan beads derived from spray-dried mulberries were stable under gastric conditions that simulated gastric fluid conditions or enhanced the bioavailability of anthocyanins [38]. In conclusion, novel technologies such as microencapsulation and HPP treatment are an effective strategy to preserve the biological functions and nutrient content of anthocyanins in food processing.

Anthocyanins comprise a subclass of phenolic phytochemicals. They are water-soluble compounds with orange, red, purple, and blue colors, and are present in fruits and vegetables such as pomegranates, berries, red grapes, purple tomatoes, and red cabbage. The most common types of anthocyanins are pelargonidin, cyanidin, peonidin, petunidin, delphinidin, and malvidin (Table 1) [21,39,40]. Anthocyanins have been investigated as bio-functional molecules possessing anti-inflammatory, antioxidant, and chemoprotective properties, and they play significant preventive roles in chronic diseases [41].

## 5. Effects of Anthocyanins in Obesity-Associated Inflammation

The abundant natural anthocyanins are well-characterized antioxidants that have been shown to eliminate reactive oxygen species (ROS) in cells, animals, and clinical studies [42,43,44,45,46,47,48].

In cell culture studies, strawberries were found to be rich in anthocyanins and contain a high antioxidant capacity. Heo and Lee (2005) showed that strawberries, as compared with bananas and oranges, dramatically reduced oxidative-stress-induced neurotoxicity in PC12 cells treated with H_2_O_2_ [42].

In animal studies, the purple sweet potato color (PSPC) protected against ROS production and restored glutathione content in high-fat diet (HFD)-induced mice [43,44]. Blackberry and blueberry anthocyanin (BLA and BBA) supplementation reduced oxidative stress and inflammation by increasing first-line defense antioxidants such as superoxide dismutase (SOD) and glutathione peroxidase (GPx). Furthermore, supplementation of BLA and BBA for 12 weeks prevented weight gain in HFD-induced obesity in C57BL/6 mice [45]. Mulberry and cherry anthocyanins reduced body weight and improved SOD and GPx activities in HFD-fed mice [46]. In clinical studies, healthy volunteers who supplemented their daily diet with 500 g of strawberries rich in anthocyanins for 1 month lowered their risk for developing CVD, as assessed by improvements in lipid profiles and antioxidant activity [47]. Li et al. (2015) studied participants with T2DM who received purified anthocyanin supplements for 24 weeks and found that anthocyanin improved lipid profiles and enhanced antioxidant capacity, as well as insulin sensitivity [48].

In summary, anthocyanins play a role as antioxidants that can eliminate ROS and help prevent chronic diseases including CVD and T2DM. Additionally, anthocyanins can reduce pro-inflammatory markers associated with obesity, such as CRP, IL-6, and TNF-α (Table 2).

### 5.1. Anti-Inflammatory Effect

Obesity has been linked to chronic low-grade systemic inflammation. Secretion of inflammatory cytokines such as IL-6 and TNF-α by adipocytes and macrophages in adipose tissue have been shown to activate the inflammatory response [64]. Alleviating inflammation can be achieved by decreasing the release of pro-inflammatory cytokines. Several studies have been conducted using cell cultures and animal models, as well as clinical trials in humans, to examine the impact of anthocyanin-rich foods on obesity-induced inflammation.

In vitro studies have shown that anthocyanin-rich fruit extracts can moderate inflammatory cytokines associated with obesity. Tart cherries rich in anthocyanins significantly reduced the expression of the IL-6 gene in lipopolysaccharide (LPS)-induced adipose stem cells [49]. Consistent with other studies, RAW264.7 mouse macrophage cells that were induced with both *Escherichia coli* LPS and recombinant interferon-c, and treated with anthocyanin-rich fractions of red raspberries at concentrations of 150 and 200 µg/mL, showed reduced nitric oxide (NO), cyclooxygenase-2 (COX-2), IL-1β, and IL-6 expression, as well as inhibition of the NF-kB inflammatory pathway [50]. Likewise, treatment with black soybean extract decreased TNF-α production, but increased adiponectin secretion and insulin sensitivity, in 3T3-L1 adipocytes [51]. In another study, sweet cherry phenolic-rich extract significantly reduced NO levels and decreased COX-2 and inducible nitric oxide synthase (iNOS) expression in LPS-induced adipose stem cells [52].

Among the animal studies, administration of a blueberry supplement containing phenolics and anthocyanins also decreased the expression of the TNF-α and IL-1β genes in HFD–fed male rats [53]. Anthocyanin-rich tart cherry extract effectively improved the pro-inflammatory cytokine profile by reducing IL-6 and leptin levels in an obese mouse model [54]. Similarly, tart cherry reduced TNF-α, IL-1β, and IL-6 expression and suppressed NF-kB inflammatory expression in HFD-induced obese rats after a 17-week intervention with tart cherry seed powder and tart cherry juice [55]. Therefore, tart cherries have the potential to prevent obesity-induced inflammation. Purified sweet cherry anthocyanins at 200 mg/kg in mice reduced body weight by approximately 11.2% and decreased the expression of the IL-6 and TNF-α genes in white adipose tissue (WAT), slowing down the progression of obesity in these mice [56]. In another study, a pomegranate peel extract (PPE) with high polyphenol content decreased COX-2 and IL-1 mRNA levels in visceral adipose tissue [57].

Anthocyanins have been shown in animal studies and cell cultures to reduce obesity-related inflammation. Therefore, many studies have aimed to evaluate the effect of anthocyanins on inflammation associated with obesity in humans. Strawberry antioxidants containing 81.65 mg anthocyanins/10 g of freeze-dried powder in a beverage significantly attenuated the postprandial inflammatory response by decreasing high-sensitivity CRP and IL-6) in an overweight population, following the consumption of a high-carbohydrate, moderate-fat meal (HCFM) [58]. Black soybean testa (BBT) extracts rich in three major anthocyanins reduced visceral fat, and improved plasma lipid profiles and inflammatory markers including TNF-α and monocyte chemoattractant protein-1 in overweight/obese adults [59]. Bhaswant et al. (2019) discovered that the high anthocyanin content in Queen Garnet plum juice significantly reduced IL-2, IL-6, IL-13, and TNF-α levels in mildly hypertensive overweight or obese people. Queen Garnet plum juice also reduced the levels of low-density lipoprotein, plasma glucose, insulin, C-peptide, leptin, and glucagon-like peptide-1 [60]. Authentic tart cherry juice (TCJ) at 240 mL of 100% TCJ (equivalent to 50 tart cherries) reduced TNF-α and monocyte chemoattractant protein-1 (MCP-1) levels compared to placebo after 4 weeks of consumption in overweight and obese individuals [61].

Both in vitro and in vivo studies have reported that foods rich in anthocyanins reduced inflammation associated with obesity, including tart cherries, PPE, and strawberry beverages. By contrast, some clinical studies found no anti-inflammatory effect of anthocyanins in people with obesity [62,63]. Zunino et al. (2012) showed that people consuming 80 g/serving of freeze-dried strawberry powder mixed with food and drinks for 3 weeks had no effect on inflammatory markers (IL-6, IL-1β, TNF-α) [62]. Similarly, consumption of commercially available red orange juice (250 mg anthocyanins/day) for 12 weeks did not show any effect on body weight or plasma inflammatory markers [63]. Possible reasons for the lack of beneficial effects of anthocyanins on obesity-induced inflammation in these studies are the use of relatively low dosages and the short length of the intervention.

### 5.2. Regulating the Gut Microbiota

Microbes living in the gut play a role in the onset of obesity [65]. Obesity is associated with changes in the gut microbiota that lead to gut dysbiosis. People with obesity have lower proportions of bacteria from the phylum Bacteroidetes, such as *Bacteroides* spp., and higher proportions from the phylum Firmicutes, such as *Bacillus* spp. and *Clostridium*. Firmicutes bacteria are distinguished by higher endotoxic activity of membrane-bound LPS compared with other gram-negative bacteria, thereby inducing more systemic inflammation. Furthermore, obesity promotes the initiation of numerous pro-inflammatory pathways by causing TLR4-mediated inflammatory responses that involve activation of the transcription factor NF-κB and the production of pro-inflammatory mediators such as IL-6, IL-1, and TNF-α [65,66,67,68]. A previous study reported that pomegranate peel extract (PPE) is rich in anthocyanins. Treatment with a PPE was shown to modulate the gut microbiota in obese mice by lowering COX-2 levels in the colon and visceral adipose tissue, as well as mRNA levels of IL-1 and IL-6 in the colon [57]. Another study in mice demonstrated that cranberry extract at 200 mg/kg reduced the abundance of *Firmicutes* but enhanced that of *Bacteroidetes*, and concluded that anthocyanin-rich cranberry extract protects dysbiosis of the microbiota in mice with diet-induced obesity [69].

### 5.3. Molecular Pathway for Effects of Anthocyanin on Obesity-Associated Inflammation

NF-κB is a family of inducible transcription factors that regulate genes involved in inflammatory responses and includes NF-κB1 (p50), NF-κB2 (p52), RelA, RelB, and c-Rel [70,71]. Under normal conditions, an NF-κB heterodimer (p50-RelA) is retained in an active form through interaction with inhibitory kappa B (IκB). The NF-κB pathway can be activated by extracellular stimuli such as ROS and a HFD. Upon stimulation, IκB is phosphorylated by the IκB kinase complex, then subsequently ubiquitinated and degraded by the 26S proteasome, allowing the p50-RelA heterodimer to translocate into the nucleus, where it promotes the expression of proinflammatory genes including TNF-α, IL-1α, and IL-1β. These genes are pathogenic in a variety of inflammatory disorders, such as obesity, T2DM, and CVD [71,72,73]. Vendramea et al. (2013) reported that an 8-week freeze-dried wild-blueberry-powder-enriched diet containing 1.5% w/w of total anthocyanin content significantly reduced the expression of genes encoding the inflammatory mediators NF-κB, TNF-α, IL-6, and CRP, both in the liver and abdominal adipose tissues, in obese Zucker rats [74]. This study concluded that anthocyanins modulated the molecular pathway of the inflammatory process, i.e., the expression of transcription factor NF-κB.

TLRs are a class of pattern recognition receptors that play a key role in the innate immune system [75]. The binding of LPS to the TLR4 receptor promotes the activation of M1 macrophages, which release pro-inflammatory cytokines [76]. Upon binding of TLR4 ligands, TLR4 recruits the adaptor molecule myeloid differentiation factor 88 (MyD88), which then recruits IL-1 receptor-associated kinase-4 (IRAK-4). Subsequently, IRAK-4 recruits TNF receptor-associated factor 6 (TRNF6) to the receptor complex. When IRAK-4 is phosphorylated, the IRAK-4–TRAF6 complex separates from the receptor complex to conjugate with transforming growth factor-β-activated kinase 1 (TAK1) [77]. Upon activation, TAK1 causes IκB to be phosphorylated and ubiquitinated, leading to degradation of IκB. As a result, the NF-κB signaling pathway is activated and inflammatory mediators are released [78]. Karunarathne et al. (2020) studied anthocyanins isolated from *Hibiscus syriacus* L. in RAW 264.7 macrophages and found that anthocyanin inhibits TLR4 in LPS-induced cells. Consequently, phosphorylation of MyD88 and IRAK4 is decreased, leading to the inhibition of the NF-κB signaling pathway [79]. Anthocyanins from black soybeans were shown to inhibit IκB phosphorylation, thereby inhibiting NF-κB nuclear translocation and activation in *Helicobacter-pylori*-induced inflammation in human gastric epithelial cells. As a result, NO and COX-2 gene expression was significantly attenuated [80]. In pro-inflammatory conditions, excess NO is converted to a pro-oxidative role, while COX-2 leads to the formation of pro-inflammatory mediators such as prostaglandin E2 [81]. These mechanisms of NF-κB pathways act as a link between oxidative stress and inflammation. Anthocyanins prevent NF-κB activation, thus suppressing the entire downstream cascade, including proinflammatory cytokines, chemokines, adhesion molecules, NO, and COX-2.

**SUMMARY POINTS**

- Obesity is associated with meta-inflammation, a newly coined term for chronic low-grade inflammation as a response to obesity.
- The state of inflammation in obesity is associated with adipose tissue dysfunction, which increases the secretion of pro-inflammatory cytokines such as TNF-α, CRP, and IL-6. Additionally, increased numbers of M1 macrophages in adipose tissue can release pro-inflammatory cytokines.
- Anthocyanins decrease the production of inflammatory cytokines in obese animal models, in studies conducted both in vitro and in vivo.
- Anthocyanins might have potential to be a treatment for obesity-related inflammation and chronic diseases.
- Be aware that the bioavailability, including metabolism and excretion, of anthocyanins is low. Therefore, future research is needed to increase the bioavailability of anthocyanins to improve their beneficial anti-inflammatory effects in people with obesity.

## 6. Conclusions

**Figure 4:**
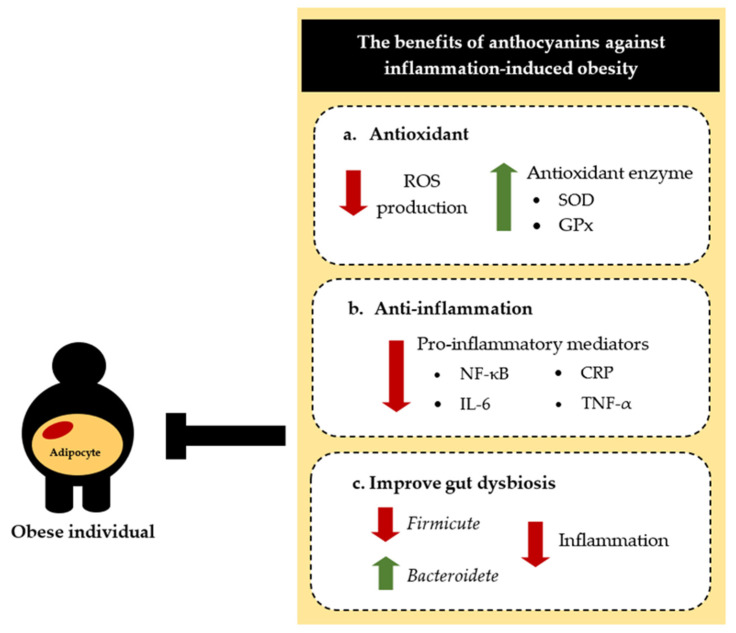
The anti-obesity effects of anthocyanins. (**a**) Anthocyanins are antioxidants that eliminate reactive oxygen species (ROS) by increasing antioxidant enzymes such as superoxide dismutase (SOD) and glutathione peroxidase (GPx). (**b**) Anthocyanins prevent nuclear factor kappa B (NF-κB) activation, thus decreasing the entire downstream cascade of pro-inflammatory mediators such as C-reactive protein (CRP), interleukin (IL)-6, and tumor necrosis factor (TNF)-α. (**c**) Anthocyanins also improve gut dysbiosis, restoring a balanced gut microbiota.

We reviewed the benefits of anthocyanin-rich foods such as sweet cherries, black soybeans, and strawberries on obesity prevention, including antioxidant and anti-inflammatory effects, and regulation of the gut microbiota in cell cultures, animal models, and human clinical trials. As shown in Figure 4, dietary anthocyanins may have anti-obesity effects and may reduce the risks of the most common chronic noncommunicable diseases.

## Figures and Tables

**Table 1:** Colors and sources of the six most well-studied anthocyanins [21,39,40].

Main Anthocyanins	Color	pH Ranges	Sources
Pelargonidin	Red, orange 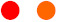	Low pH (pH < 3)	Radish, pomegranate, red potato, ripe raspberry
Cyanidin	Red, reddish-purple 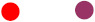	Low to neutral pH (pH 3–7)	Blackberry, red sweet potato, purple corn, tart and sweet cherry
Peonidin	Purplish-red 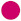	Neutral pH (pH 6–7)	Sweet potato, cranberry, grape, purple corn
Petunidin	Purple, dark red 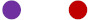	Low to high pH (pH 3–8)	Blackcurrant, black bean, red berry
Delphinidin	Purple, blue-reddish 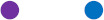	Neutral to high pH (pH 7–11)	Pomegranate, black bean, purple tomato
Malvidin	Purple 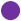	Neutral pH (pH 7–8)	Blueberry, red wine, bilberry, mulberry

**Table 2:** Effects of anthocyanins on obesity-associated inflammation [49,50,51,52,53,54,55,56,57,58,59,60,61,62,63].

Food Source	Bioactive of Anthocyanins	Effects	Study Group	Reference
Tart cherries	Cyanidin-3-*O*-glucoside Cyanidin-3-*O*-glucosyl-rutinoside Cyanidin-3-*O*-rutinoside	Reduced IL-6 level	Adipose stem cells	[49]
Red raspberries	Identified anthocyanins N/A	Reduced NO, COX-2, IL-1β, IL-6 expression Suppressed NF-kB pathway	RAW264.7 macrophages	[50]
Black soybeans	Cyanidin-3-*O*-glucoside Pelargonidin 3-glucoside Delphinidin-3-glucoside	Reduced TNF-α secretion Increased adiponectin and insulin sensitivity	3T3-L1 cells	[51]
Sweet cherry	Cyanidin 3-*O*-rutinoside	Reduced NO level Decreased COX-2 and iNOS expression	RAW 264.7 macrophages	[52]
Blueberry supplementation	Phenolics and anthocyanin	Improved TNF-α and IL-1β gene expression Improved gut microbiota	Male Wistar rats fed HFD	[53]
Tart cherry extract	Cyanidin-3-*O*-glucoside Cyanidin-3-*O*-glucosyl-rutinoside Cyanidin-3-*O*-rutinoside	Decreased IL-6 andleptin levels	Obese mice	[54]
Tart cherry seed powder and tart cherry juice	Identified anthocyanins N/A	Reduced TNF-α, IL-1β and IL-6 expression Suppressed NF-kB pathway	Male Wistar rats fed HFD	[55]
Sweet cherry	Cyanidin-3-*O*-glucosyl-rutinoside Cyanidin-3-*O*-rutinoside Pelargonidin 3-rutinoside	Decreased body weight Decreased IL-6 and TNF-α gene expression in WAT	Male C57BL/6 mice fed HFD	[56]
Pomegranate peel extract	Ellagitannins and anthocyanins	Reduced COX-2 in the colon and visceral adipose tissue Reduced mRNA levels of IL-1β in the visceral adipose tissue Reduced IL-6 in the colon	Balb/c obese mice	[57]
Strawberrybeverage	Pelargonidin 3-glucoside Pelargonidin 3-malonylglucoside Pelargonidin 3-rutinoside	Improved hs-CRPand IL-6	Overweight adults	[58]
Black soybean testa extracts	Cyanidin-3-glucoside Delphinidin-3-glucoside Petunidin-3-glucoside	Improved visceral fat Improved plasma Lipid profiles Reduced TNF-α and MCP-1	Overweight and obese adults	[59]
Queen garnet plum	Cyanidin 3-*O*-β-D-glucoside	Reduced LDL level, plasma glucose, insulin, C-peptide, leptin and GLP-1 concentrations	Mildly hypertensive obese/overweight adults	[60]
Authentic tart cherry juice	Cyanidin-3-2G-glucosylrutinoside Cyanidin-3-glucoside Cyanidin-3-rutinoside Peonidin-3-rutinoside	Reduced TNF-α and MCP-1	Overweight and obese adults	[61]
Strawberry	Identified anthocyanins N/A	No effect on inflammatory markers (e.g., IL-6, IL-1β, TNF-α)	Obese healthy males and females	[62]
Commercially available red orange juice	Identified anthocyanins N/A	No effect on body weight and plasma inflammatory markers	Overweight or obese females	[63]

NO = nitric oxide; iNOS = inducible nitric oxide synthase; COX-2 = cyclooxygenase-2; IL = interleukin; NF-kB = nuclear factor-kB; TNF = tumor necrosis factor; HFD = high-fat diet; N/A = not applicable; hs-CRP = high-sensitivity C-reactive protein; MCP-1 = monocyte chemoattractant protein-1; LDL = low-density lipoprotein; GLP-1 = glucagon-like peptide 1.
